# The Influence of Past Metronidazole Exposure on the Outcome of *Helicobacter pylori* Eradication

**DOI:** 10.3389/fmicb.2022.857569

**Published:** 2022-03-25

**Authors:** Younghee Choe, Joon Sung Kim, Hyun Ho Choi, Dae Bum Kim, Jae Myung Park, Jung Hwan Oh, Tae Ho Kim, Dae Young Cheung, Woo Chul Chung, Byung-Wook Kim, Sung Soo Kim

**Affiliations:** ^1^Division of Gastroenterology and Hepatology, Department of Internal Medicine, Incheon St. Mary’s Hospital, College of Medicine, Catholic University of Korea, Seoul, South Korea; ^2^Division of Gastroenterology and Hepatology, Department of Internal Medicine, Uijeongbu St. Mary’s Hospital, College of Medicine, Catholic University of Korea, Seoul, South Korea; ^3^Division of Gastroenterology and Hepatology, Department of Internal Medicine, St. Vincent’s Hospital, College of Medicine, Catholic University of Korea, Seoul, South Korea; ^4^Division of Gastroenterology and Hepatology, Department of Internal Medicine, Seoul St. Mary’s Hospital, College of Medicine, Catholic University of Korea, Seoul, South Korea; ^5^Division of Gastroenterology and Hepatology, Department of Internal Medicine, Eunpyeong St. Mary’s Hospital, College of Medicine, Catholic University of Korea, Seoul, South Korea; ^6^Division of Gastroenterology and Hepatology, Department of Internal Medicine, Bucheon St. Mary’s Hospital, College of Medicine, Catholic University of Korea, Seoul, South Korea; ^7^Division of Gastroenterology and Hepatology, Department of Internal Medicine, Yeouido St. Mary’s Hospital, College of Medicine, Catholic University of Korea, Seoul, South Korea

**Keywords:** anti-bacterial agents, bismuth quadruple therapy, microbial drug resistance, duration of therapy, eradication rate, *Helicobacter* infections, metronidazole

## Abstract

**Background:**

Bismuth quadruple therapy (BQT) is recommended as empirical first-line therapy because it is not affected by antibiotic resistance. We examined whether past exposure to metronidazole affected BQT outcomes.

**Methods:**

The records of seven hospitals were searched for patients who received BQT for *Helicobacter pylori* eradication between 2009 and 2020. The association between past metronidazole exposure and the eradication rate was evaluated.

**Results:**

This study was a multicenter retrospective study. Around 37,602 people tested for *H. pylori* infection were identified, and 7,233 received BQT. About 2,802 (38.7%) underwent a 13C-urea breath test to confirm eradication. The BQT efficacy was 86.4% among patients without metronidazole exposure and 72.8% among patients with exposure (*p* < 0.001). The eradication rate of BQT 14 days in patients with past exposure was higher than that of BQT <14 days (85.5 vs. 66.0%, *p* = 0.009). Multivariate analysis revealed that past metronidazole exposure [odds ratio (OR) 2.6, 95% CI 1.8–3.7; *p* < 0.001] and BQT <14 days (OR 1.5, 95% CI 1.2–2.0; *p* = 0.002) were independent risk factors for eradication failure.

**Conclusion:**

Past metronidazole exposure significantly lowered the BQT eradication rate. BQT 14 days should be recommended for patients with suspected metronidazole exposure.

## Introduction

*Helicobacter pylori* is the primary etiologic cause of gastric adenocarcinoma (Lee et al., 2016; Hooi et al., 2017; Chiang et al., 2021). A recent systematic review reported that eradication resulted in a 46% reduced incidence of and 39% reduced mortality from gastric cancer (de Martel et al., 2020; Ford et al., 2020). The Kyoto global consensus and Houston consensus conferences recommend treating all individuals with proven *H. pylori* infection (Sugano et al., 2015; El-Serag et al., 2018). Traditionally, eradication for *H. pylori* was based on a standard triple regimen consisting of a proton pump inhibitor (PPI), amoxicillin, and clarithromycin. However, this regimen is no longer recommended without susceptibility testing due to the high rates of clarithromycin resistance in many countries (Malfertheiner et al., 2011; Yeo et al., 2018).

Many current guidelines recommend bismuth quadruple therapy (BQT) as first-line therapy in areas with high clarithromycin resistance because BQT is less affected by antibiotic resistance (Fallone et al., 2016; Chey et al., 2017; Malfertheiner et al., 2017; Chen et al., 2019; Jung et al., 2020; Shah et al., 2021). However, BQT is difficult to use due to its frequent dosing, side effects, and availability (Howden and Graham, 2021). According to the recent European Registry, BQT users reported higher rates of side effects than those on other regimens (Nyssen et al., 2021a). BQT is generally recommended for 14 days rather than 7 or 10 days (Fallone et al., 2019; Kim et al., 2020; Shah et al., 2021). This is because prolonging the duration of metronidazole use to 14 days is expected to overcome resistance. However, few studies have confirmed this in clinical trials.

Several studies reported that clarithromycin-based triple therapy was affected by past macrolides exposure (Boltin et al., 2019; Kwon et al., 2019). There have been studies examining the relationship between past metronidazole exposure and BQT eradication rates. However, no meaningful additional analysis was performed other than the eradication rate due to the small number of patients (McNulty et al., 2012; Boltin et al., 2020; Lee et al., 2020). Our purpose was to investigate whether past metronidazole exposure influenced the eradication rates of the BQT regimen. We also examined the eradication rates according to the BQT duration in patients with or without metronidazole exposure.

## Materials and Methods

### Study Design and Data Extraction

This was a multicenter retrospective study of patients from seven university hospitals from 2009 to 2020. We identified patients aged 20–79 years who underwent an endoscopy and were diagnosed with *H. pylori* by histology or a rapid urease test. Patients who were prescribed BQT for eradication were included. Age, sex, body mass index, smoking history, endoscopic findings, and pathologic results were identified. We also investigated the BQT duration and the type of PPI used for the BQT regimen. Medical records were reviewed to identify patients who had been prescribed metronidazole before their BQT prescription.

This study protocol was approved by the Institutional Review Board (IRB) of Catholic Medical Center (IRB approval number, XC20WIDI0119). The requirement for written informed consent was waived because anonymous data were used. This study followed the ethical principles of the Declaration of Helsinki.

### Past Metronidazole Exposure

Patients’ medical records were examined to determine whether they had received metronidazole before the prescription of BQT. All inpatient or outpatient antibiotic prescription records were searched, and metronidazole prescription records were extracted. The patient’s diagnosis, drug administration method, dosage, the number of doses per day, and total administration duration were examined together with all metronidazole prescription records. However, only the intravenous or oral administration cases were extracted, and topical preparations were excluded. We also investigated past metronidazole exposure intervals up to the BQT therapy regimen.

### Confirmation of *Helicobacter pylori* Infection and Eradication Regimen

*Helicobacter pylori* infection was confirmed by rapid urease test, histopathology, or urea breath test. Dual priming oligonucleotide (DPO)-based multiplex PCR (Seeplex® ClaR-*H. pylori* ACE detection kit, Seegene Inc., Seoul, Korea) was performed in some patients confirmed with *H. pylori* infection to examine the presence of clarithromycin resistance. The precise methods of DPO-PCR have been described elsewhere (Posteraro et al., 2006; Woo et al., 2009; Lehours et al., 2011). BQT was prescribed as second-line therapy after the failure of the first-line triple therapy or first-line therapy in patients confirmed with clarithromycin resistance by DPO-PCR methods.

Bismuth quadruple therapy consisted of a PPI (standard dose) twice daily, bismuthate tripotassium dicitrate (300 mg) and tetracycline (500 mg) four times daily, and metronidazole (500 mg) three times daily. The PPIs used were lansoprazole (30 mg/T), pantoprazole (40 mg/T), rabeprazole (20 mg/T), esomeprazole (40 mg/C), and ilaprazole (10 mg/T). The BQT duration was 7, 10, or 14 days at the physician’s discretion. All patients underwent a urea breath test after 4–12 weeks to confirm the success of eradication. For the urea breath test, 13C-urea 100 mg tablets (UBiTkit™, Otsuka Electronics, Co., Ltd., Osaka, Japan) were orally taken with water after fasting for at least 4 h. Exhaled breath samples obtained after that were analyzed using the 13C-urea breath test (UBiT-IR300®), and the cut-off value for judging *H. pylori*-positive and negative was set to 2.5‰ (Graham et al., 1987; Graham and Klein, 1991; Atherton and Spiller, 1994).

### Endpoints and Statistical Analysis

The primary endpoint was to compare the BQT eradication rates according to patients’ past metronidazole exposure. The secondary endpoint was to identify risk factors associated with eradication failure. We examined whether past metronidazole exposure influenced the BQT eradication rate and whether prolonging the BQT duration was associated with treatment outcomes. We also examined whether the duration of past metronidazole exposure affected the BQT outcome and whether the interval between the past exposure and the prescription of BQT affected *H. pylori* eradication rates.

The baseline patient characteristics are summarized using descriptive statistics. Continuous data are presented as the mean (SD) or median (interquartile range), and categorical data are given quantities and proportions. The student’s *t*-test was used to compare continuous variables. Categorical variables were compared using the *χ*^2^ test or Fisher’s exact test. The duration of the past treatment was analyzed by linear-by-linear association because the independent variables were classified to be three or more. Logistic regression analysis was used to identify independent risk factors for eradication failure, and multiple regression analysis was used to analyze the correlation between these factors and eradication failure (i.e., factors that showed significant differences in univariate analysis).

The SPSS statistical program, version 22 (SPSS, Chicago, Illinois), was used for all analyses, and the significance level was set at a value of *p* < 0.05.

## Results

### Baseline Characteristics

**Figure 1:**
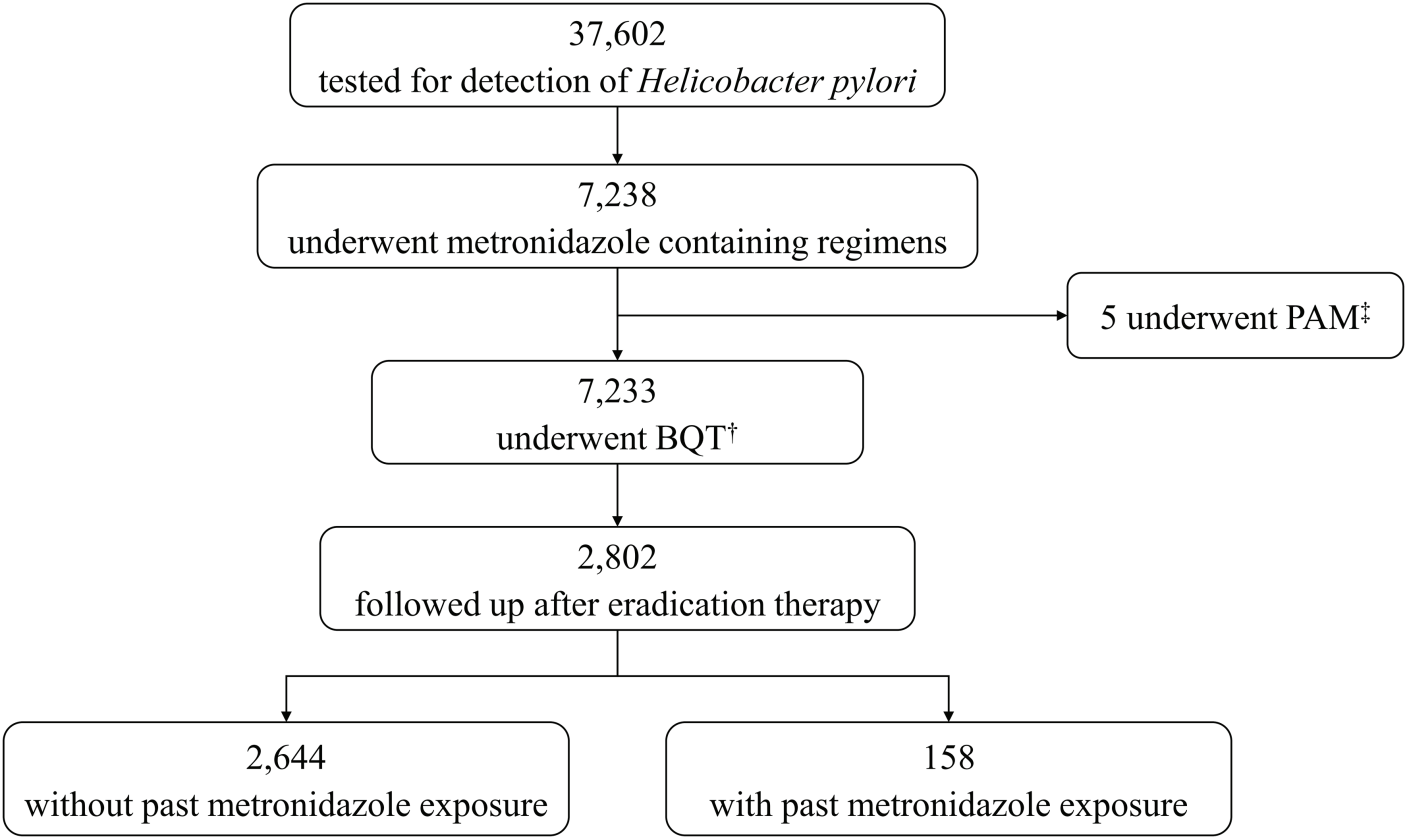
Flow chart. ^†^BQT, bismuth quadruple therapy, ^‡^PAM, proton pump inhibitor, amoxicillin, and metronidazole.

Around 37,602 patients were tested for *H. pylori* infection in the seven participating hospitals, and 7,238 of them received metronidazole containing eradication therapy. In total, 7,233 patients were prescribed BQT. Of the 7,233 patients prescribed BQT, 2,802 were followed up after eradication and included in this study (Figure 1). The mean age was 57.8 ± 11.4 years, and 53.2% of patients were male. About 158 (5.6%) patients had previously received metronidazole intravenously or orally. The baseline characteristics of the groups with and without past metronidazole exposure are shown in Table 1.

**Table 1:** Baseline characteristics.

	Total (*N* = 2,802)	Without metronidazole exposure (*N* = 2,644)	With metronidazole exposure (*N* = 158)	*p*-value
Age, years	57.8 ± 11.4	57.9 ± 11.4	56.3 ± 11.0	0.085
Male sex	1,492/2,802 (53.2)	1,415/2,644 (53.5)	77/158 (48.7)	0.242
BMI	24.4 ± 3.4	24.4 ± 3.4	24.0 ± 3.2	0.210
Current smoker	325/1,787 (11.6)	307/1,669 (18.4)	18/118 (15.3)	0.498
Order of BQT				
First-line^†^	241/2,802 (8.6)	239/2,644 (9.0)	2/158 (1.3)	<0.001
Second-line	2,561/2,802 (91.4)	2,405/2,644 (91.0)	156/158 (98.7)	
Type of PPI				
Lansoprazole	963/2,802 (34.4)	900/2,644 (34.0)	63/158 (39.9)	0.657
Pantoprazole	844/2,802 (30.1)	818/2,644 (30.9)	26/158 (16.5)	
Rabeprazole	801/2,802 (28.6)	736/2,644 (27.8)	65/158 (41.1)	
Esomeprazole	128/2,802 (4.6)	125/2,644 (4.7)	3/158 (1.9)	
Ilaprazole	66/2,802 (2.4)	65/2,644 (2.5)	1/158 (0.6)	
BQT duration				
7 days	1,796/2,802 (64.1)	1,705/2,644 (64.5)	91/158 (57.6)	0.115
10 days	233/2,802 (8.3)	221/2,644 (8.4)	12/158 (7.6)	
14 days	773/2,802 (27.6)	718/2,644 (27.2)	55/158 (34.8)	

Data represent the number of patients (%) or mean ± SD.

† Clarithromycin resistance.

BMI, body mass index; BQT, bismuth quadruple therapy; and PPI, proton pump inhibitor.

Bismuth quadruple therapy regimen was prescribed as second-line therapy in 2,561 (91.4%) patients and as first-line therapy in 241 (8.6%) patients. BQT was prescribed more frequently as a first-line regimen in patients without metronidazole exposure (*p* < 0.001). The BQT duration was 7 days in 64.1%, 10 days in 8.3%, and 14 days in 27.6%. There was no significant difference in the duration of BQT in patients with or without metronidazole exposure (*p* = 0.115).

### Past Metronidazole Exposure and *Helicobacter pylori* Eradication Rate

The BQT eradication rates for *H. pylori* were 86.4% in patients without metronidazole exposure and 72.8% in patients with exposure (*p* < 0.001; Table 2). There was no significant difference in the eradication rates of BQT <14 days and 14 days in patients without metronidazole exposure (87.5 vs. 89.1%, *p* = 0.289). The eradication rates of BQT 14 days were higher than BQT <14 days in patients with exposure (85.5 vs. 67.0%, *p* = 0.014).

**Table 2:** Eradication rates of bismuth quadruple therapy for *Helicobacter pylori* with and without past metronidazole exposure.

	Total	Without metronidazole exposure (*N* = 2,644)	With metronidazole exposure (*N* = 158)	*p*-value
Eradication rates	2,399/2,802 (85.6)	2,284/2,644 (86.4)	115/158 (72.8)	<0.001
BQT <14 days	1,711/2,029 (84.3)	1,643/1,926 (85.3)	68/103 (66.0)	<0.001
BQT 14 days	688/773 (89.0)	641/718 (89.3)	47/55 (85.5)	0.383

Data represent the number of patients (%). BQT, bismuth quadruple therapy.

### Eradication Rates According to the Duration of Past Metronidazole Exposure and Interval to BQT

**Figure 2:**
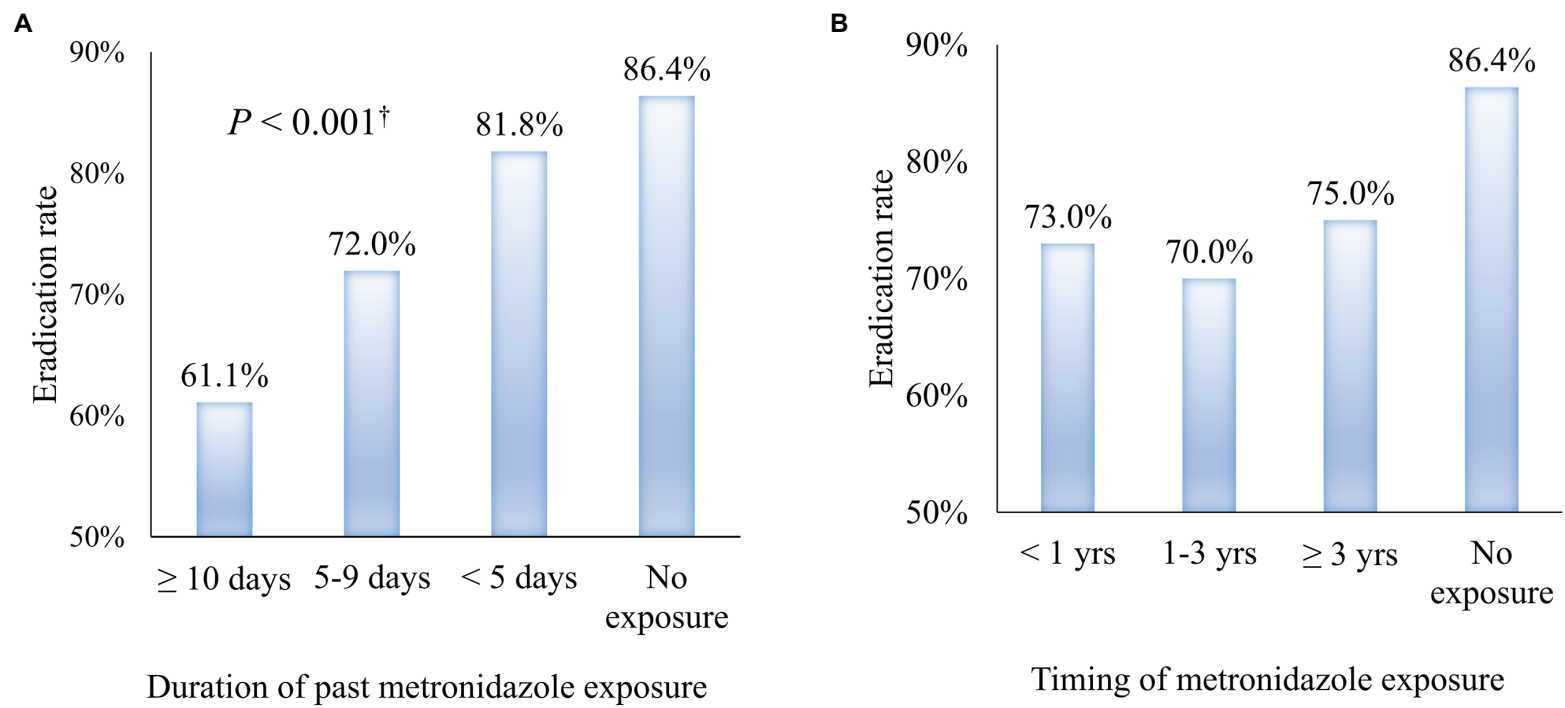
The trend of *Helicobacter pylori* eradication rate according to past metronidazole exposure. **(A)** Eradication rate of *H. pylori* by the duration of previous metronidazole exposure **(B)** Eradication rate of *H. pylori* by the interval from past metronidazole exposure to the prescription of bismuth quadruple therapy.

We examined whether the duration of past metronidazole exposure and interval to BQT affected eradication. The eradication rates for less than 5 days, 5–9 days, and more than 10 days of past exposure were 81.8, 72.0, and 61.1% (Table 3). Figure 2A showed a tendency for the eradication rate to increase as the metronidazole exposure duration in the past increases (*p* < 0.001 by linear-by-linear association).

**Table 3:** Past metronidazole exposure duration in people receiving bismuth quadruple therapy.

	Total number of patients	Number of patients successfully eradicated	Ratio
Metronidazole exposure duration, days			
Median (range)	5 (1–19)		
0	2,644	2,284	86.4%
1–4	33	27	81.8%
5–9	108	77	71.3%
≥10	17	11	64.7%

Value of *p* < 0.001 analyzed according to linear-by-linear association according to exposure duration.

We also examined if the interval from past exposure to BQT prescription affected its outcomes. The intervals between past metronidazole exposure were 73, 70, and 75% for patients less than 12 months, 12–36 months, and ≥36 months, respectively. There was no significant difference between the exposure interval and the current BQT eradication rate (Figure 2B).

### Univariate and Multivariate Analysis of Factors Related to *Helicobacter pylori* Eradication Failure

We performed a logistic regression analysis to identify *H. pylori* eradication failure (Table 4). Univariate analysis revealed BQT prescribed as first-line, past metronidazole exposure and BQT <14 days to be significantly associated with eradication failure. Multivariate analysis confirmed that BQT prescribed as first-line (odds ratio 1.79; 95% CI, 1.28–2.50; *p* = 0.001), past metronidazole exposure (odds ratio 2.57; 95% CI, 1.77–3.73; *p* < 0.001), and BQT <14 days (odds ratio 1.51; 95% CI, 1.16–1.96; *p* = 0.002) were independent risk factors for *H. pylori* eradication failure.

**Table 4:** Univariate and multivariate analysis of factors related to *Helicobacter pylori* eradication failure.

	Univariate analysis		Multivariate analysis	
	Odds ratio (95% CI)	*p*-value	Odds ratio (95% CI)	*p*-value
Age ≥ 60 years	0.88 (0.71–1.09)	0.248		
Male	0.99 (0.80–1.23)	0.949		
BMI ≥ 25 kg/m^2^	1.04 (0.79–1.37)	0.790		
Current smoker	1.01 (0.87–1.17)	0.942		
Past history of gastric cancer	1.14 (0.39–3.32)	0.817		
Prescribed BQT for the 1st line	1.88 (1.36–2.59)	<0.001	1.79 (1.28–2.50)	0.001
Past metronidazole exposure	2.37 (1.64–3.43)	<0.001	2.57 (1.77–3.73)	<0.001
BQT <14 days	1.50 (1.17–1.94)	0.002	1.51 (1.16–1.96)	0.002

BMI, body mass index; BQT, bismuth quadruple therapy; and CI, confidence interval.

## Discussion

Current guidelines recommend BQT as empirical therapy because it has proven to be effective in areas with high antibiotic resistance (Fallone et al., 2016; Chey et al., 2017; Malfertheiner et al., 2017; Chen et al., 2019; Shah et al., 2021). Metronidazole resistance is high in many countries, and a recent study reported global resistance rates to be 40–70% (Savoldi et al., 2018; Schubert et al., 2020; Megraud et al., 2021). BQT 14 days is generally recommended in areas with high metronidazole resistance as extending the duration to 14 days is expected to overcome resistance. In our clinical practice, patients often complain of difficulty taking BQT for 14 days due to the complexity of the regimen and adverse events. According to a European Registry survey from 2013 to 2018, patients prescribed BQT had the highest risk of adverse events compared with other eradication regimens (Nyssen et al., 2021b). A systematic review reported that adverse events increased as the BQT duration prolonged from 7 to 14 days (Yuan et al., 2013). These studies suggest that the dosage frequency and adverse events of BQT can affect patient compliance. There is also little evidence that increasing BQT duration to 14 days is more effective than 7 or 10 days in patients with metronidazole resistance.

Metronidazole resistance is not easy to demonstrate. Culture tests can confirm antibiotic resistance. However, *H. pylori* are intricate to incubate, and the long time deters its use in clinical practice. The E-test, widely used to measure antibiotic resistance, is known to overestimate metronidazole resistance (Osato et al., 2001). An *in vitro* test for metronidazole resistance is not an absolute preclusion criterion for use because *in vitro* resistance does not reliably correlate with *H. pylori* eradication failure. A recent study reported no association between metronidazole resistance determined by molecular-based experiments and eradication success (Argueta et al., 2021). Phenotypic methods for metronidazole resistance testing are not well standardized, and results can vary depending on the method used, which can result in lower predictive value.

In our study, we found that past metronidazole exposure lowered the BQT eradication rate. Prolonging the BQT duration to 14 days significantly increased eradication rates in patients with past exposure. The eradication rates of BQT <14 days in patients without exposure were similar to BQT 14 days. This suggest that BQT regimens of shorter duration may be effective in patients without metronidazole exposure history or metronidazole resistance. For patients suspected with a past metronidazole exposure, BQT should be prescribed for 14 days or levofloxacin-based regimens may be considered in areas with low quinolone resistance.

A previous study investigated the relationship between the dose of past macrolides exposure and the eradication rate of standard triple therapy (Boltin et al., 2019). They reported that increased macrolides exposure was associated with lower eradication rates. To our knowledge, no study examined if the duration of past metronidazole exposure and the interval from exposure to eradication affected the eradication rate. In our study, the eradication rate decreased as the duration of past metronidazole exposure increased. However, the interval from metronidazole exposure to BQT prescription was not related to the eradication rate. Based on our results, BQT 14 days may be recommended for patients with suspected past metronidazole exposure, regardless of the interval from exposure. BQT 7 days can achieve high eradication rates with fewer side effects in areas with low metronidazole resistance or in patients without metronidazole exposure.

A three-in-one formulation (Pylera®) of three drugs, bismuth subcitrate, metronidazole, and tetracycline, is frequently prescribed in the United States and Europe. This formulation is preferred to BQT because of its small number of capsules but consists of 10 days. Few studies have compared the BQT eradication rates for 10 and 14 days (Dore et al., 2011). According to our study, 10-day BQT may not be sufficient in patients with past metronidazole exposure, suggesting that 10-day regimens should be avoided in areas with high metronidazole resistance.

Our study has several limitations. First, our study is a retrospective study which inevitably includes inherent limitations due to its study design. Most importantly, the baseline characteristics of patients with and without exposure were different. Specifically, BQT was prescribed as first-line regimens more often in patients without past metronidazole exposure. Second, patients classified as unexposed in this study might have been prescribed metronidazole at other hospitals. However, it is essential to note that metronidazole is not commonly prescribed in primary clinics in Korea (Park et al., 2017; Health Insurance Review and Assessment Service, 2021). Also, reclassification of these patients into the exposed group is expected to strengthen our findings further. Third, the follow-up loss rate was high in patients who were prescribed BQT. However, our study was retrospective, and the high follow-up loss rate reflects the reality of actual clinics. Another study reported that only 34.9% of those prescribed *H. pylori* eradication regimens visited the hospital to check their eradication results (Kumar et al., 2021). Finally, this study was conducted in a region with high metronidazole resistance. Our results may not be applicable in areas with different metronidazole.

In conclusion, past metronidazole exposure lowered the *H. pylori* eradication rate of BQT regimens, and longer exposure duration was associated with lower eradication rates. Prolonging the BQT duration to 14 days should be considered in patients with past metronidazole exposure.

## Data Availability Statement

The original contributions presented in the study are included in the article/supplementary material, further inquiries can be directed to the corresponding author.

## Ethics Statement

The studies involving human participants were reviewed and approved by Institutional Review Board (IRB) of Catholic Medical Center (IRB approval number, XC20WIDI0119). Written informed consent for participation was not required for this study in accordance with the national legislation and the institutional requirements.

## Author Contributions

YC designed the study, reviewed the literature, acquired and analyzed data, administrated the project, performed statistical analyses, wrote the original draft, and reviewed and edited the manuscript. JK supervised the study and was responsible for paper conception and manuscript review, and editing. JP acquired data and reviewed and edited the manuscript. HC, DK, JO, TK, and DC acquired data and edited the manuscript. WC, B-WK, and SK analyzed data and edited the manuscript. All authors contributed to the article and approved the submitted version.

## Conflict of Interest

The authors declare that the research was conducted in the absence of any commercial or financial relationships that could be construed as a potential conflict of interest.

## Publisher’s Note

All claims expressed in this article are solely those of the authors and do not necessarily represent those of their affiliated organizations, or those of the publisher, the editors and the reviewers. Any product that may be evaluated in this article, or claim that may be made by its manufacturer, is not guaranteed or endorsed by the publisher.

## Abbreviations

BQT: Bismuth quadruple therapy
DPO: Dual-priming oligonucleotide
*H. pylori*: *Helicobacter pylori*
PCR: Polymerase chain reaction
PPI: Proton pump inhibitor
